# A Powerful Disinfectant

**Published:** 1865

**Authors:** 


A POWERFUL DISINFECTANT.—The fumes of burning coffee have long been known to act as a powerful disinfectant. Experiments have recently been made in Paris to prove this. A quantity of meat was hung up in a closed room until decomposed, and then a chafing-dish was introduced, and five hundred grammes of coffee thrown on the fire. In a few minutes the room was completely disinfected. In another room sulphurretted hydrogen and ammonia were developed, and ninety grammes of coffee destroyed the smell in about a half a minute.

